# Supplementary material for: Parallels and contrasts between the cnidarian and bilaterian maternal-to-zygotic transition are revealed in Hydractinia embryos
Source: bioRxiv. 2023 May 10:2023.05.09.540083. Preprint. [Version 1] doi: 10.1101/2023.05.09.540083 (PMC10197650; doi:10.1101/2023.05.09.540083)
Supplement: 1 [file NIHPP2023.05.09.540083V1-supplement-1.pdf]

bioRxiv preprint doi: https://doi.org/10.1101/2023.05.09.540083; this version posted May 10, 2023. The copyright holder for this preprint (which was not certified by peer review) is the author/funder, who has granted bioRxiv a license to display the preprint in perpetuity. It is made available under aCC-BY 4.0 International license.

Supplementary Figures

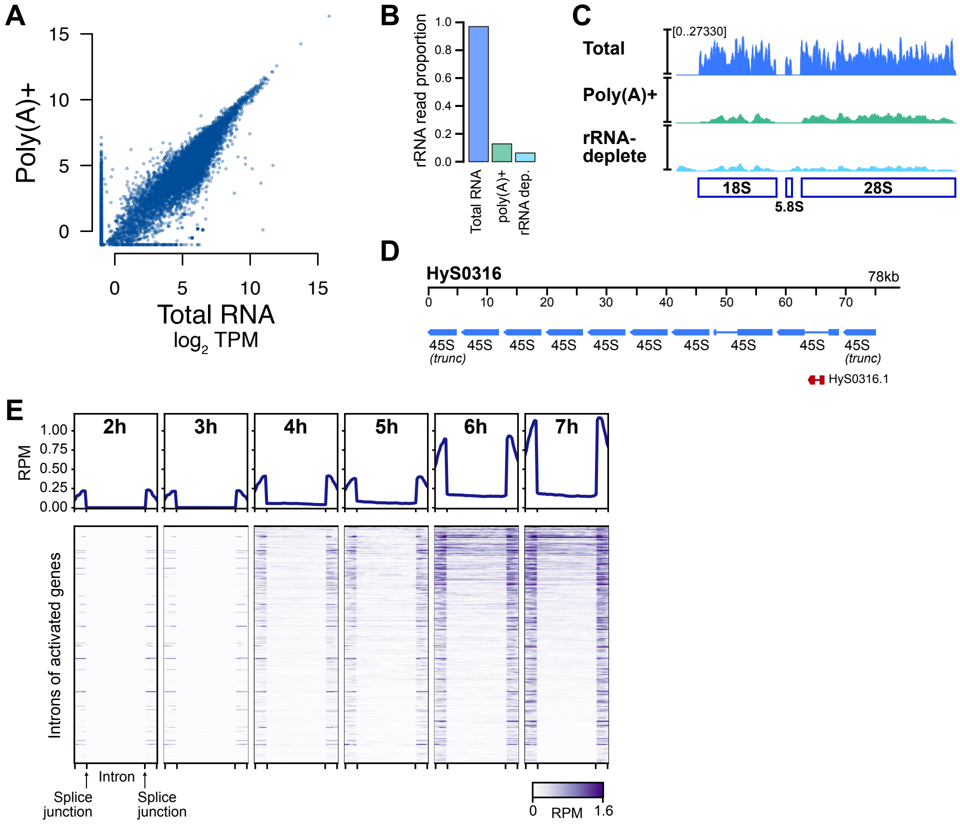

Supplementary Figure S1. rRNA-depletion RNA-seq facilitates detection of genome activation. (A) Biplot comparing total RNA-seq (no selection) to poly(A)+ selected RNA-seq. **(B)** Proportion of sequencing reads mapping to rRNA without selection, with poly(A)+ selection, and with rRNA depletion at 1 hour post fertilization (h.p.f.). **(C)** Browser tracks over the composite 45S rRNA locus showing RNA-seq coverage in the different selection strategies. **(D)** Browser track showing a predicted array of 45S genes on the genome scaffold HyS0316. **(E)** Heatmaps showing intronic RNA-seq coverage of activated genes over time. RPM = reads per million.

bioRxiv preprint doi: https://doi.org/10.1101/2023.05.09.540083; this version posted May 10, 2023. The copyright holder for this preprint (which was not certified by peer review) is the author/funder, who has granted bioRxiv a license to display the preprint in perpetuity. It is made available under aCC-BY 4.0 International license.

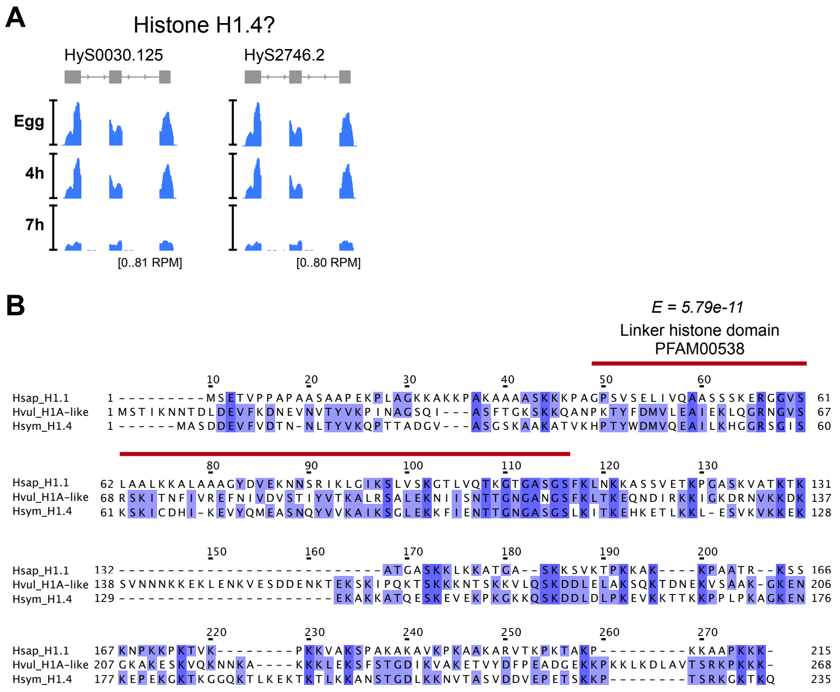

Supplementary Figure S2. ***H. symbiolongicarpus*** **may encode a novel histone H1. (A)** Browser tracks showing two identical novel H1 genes (H1.4) and RNA-seq coverage over time. **(B)** Multiple alignment of the amino acid sequences of human H1.1 (top), *Hydra vulgaris* H1A-like (middle) and the novel *H. symbiolongicarpus* H1.4 (bottom). The CD-Search annotated linker histone domain is marked in red.

bioRxiv preprint doi: https://doi.org/10.1101/2023.05.09.540083; this version posted May 10, 2023. The copyright holder for this preprint (which was not certified by peer review) is the author/funder, who has granted bioRxiv a license to display the preprint in perpetuity. It is made available under aCC-BY 4.0 International license.

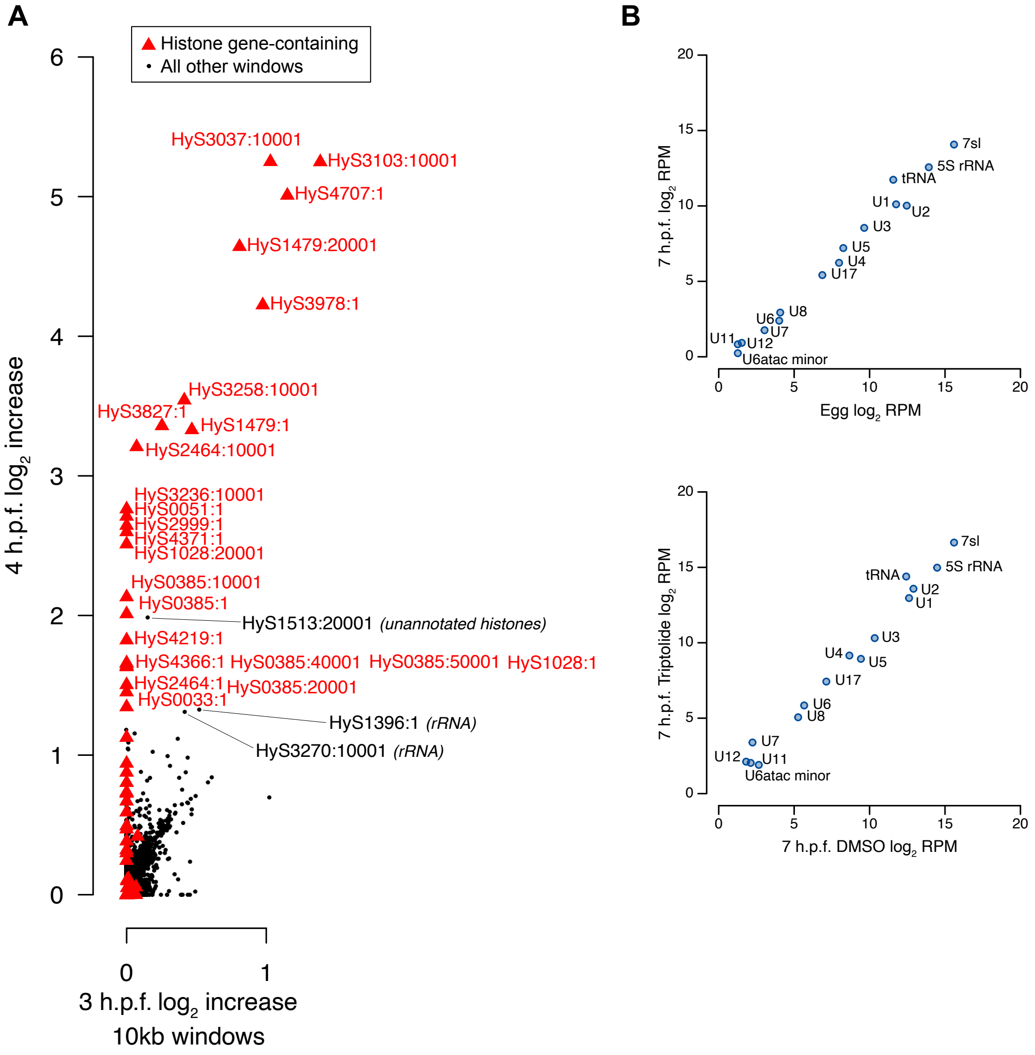

Supplementary Figure S3. Unannotated expressed genes are mostly non-coding. (A) Comparison of activation levels of 10-kb windows tiled across the genome at 3 hours post fertilization (h.p.f.) versus egg (x axis) and 4 h.p.f. versus egg (y axis). Windows with major levels of activation are accounted for by histone genes (red triangles), except for two windows that have predicted ribosomal RNA genes. **(B)** Biplots of summed expression across the predicted noncoding gene classes uncovered by Stringtie transcriptome assembly.

bioRxiv preprint doi: https://doi.org/10.1101/2023.05.09.540083; this version posted May 10, 2023. The copyright holder for this preprint (which was not certified by peer review) is the author/funder, who has granted bioRxiv a license to display the preprint in perpetuity. It is made available under aCC-BY 4.0 International license.

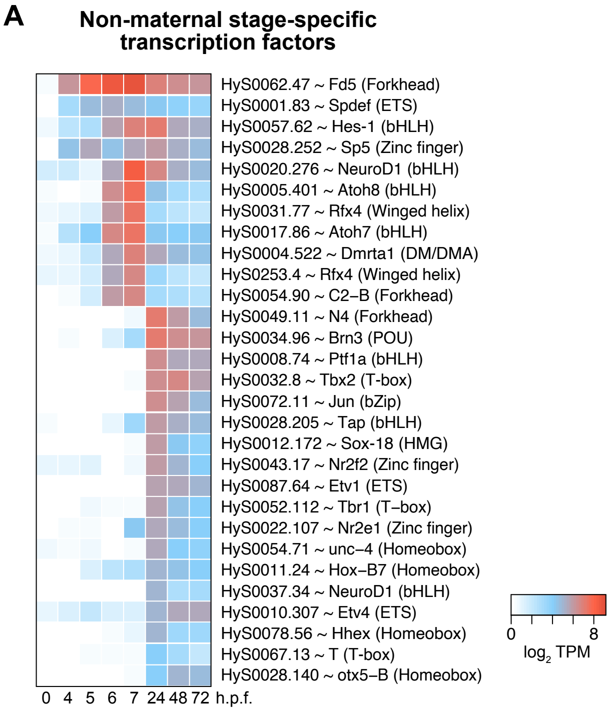

Supplementary Figure S4. Stage-specific transcription factors are expressed after genome activation.
(A) Heatmap showing expression patterns of non-maternal stage-specific transcription factors. Gene names are the best BLAST hit to UniProtKB / SwissProt, DNA binding domains are in parentheses.

bioRxiv preprint doi: https://doi.org/10.1101/2023.05.09.540083; this version posted May 10, 2023. The copyright holder for this preprint (which was not certified by peer review) is the author/funder, who has granted bioRxiv a license to display the preprint in perpetuity. It is made available under aCC-BY 4.0 International license.

A

		Best SwissProt Hit			
**Gene ID**	**Factor**	**Accession**	**Species**	**E-value**	**Egg TPM**
HyS0013.274	CNOT1	A1A5H6.1	*D. rerio*	0	36.1
HyS0005.44	CNOT2	Q9NZN8.1	*H. sapiens*	3.7E-98	131.3
HyS0006.62	CNOT3	Q8K0V4.1	*M. musculus*	9.8E-95	60.8
HyS0049.13	CNOT4	O95628.3	*H. sapiens*	1.4E-120	54.8
HyS0018.317	CCR4/CNOT6	Q9ULM6.2	*H. sapiens*	1.2E-179	102.5
HyS0005.193	CAF1/Pop2/CNOT7	Q3ZC01.1	*B. taurus*	1.6E-144	85.9
HyS0003.398	CAF40/CNOT9	Q6IP65.1	*X. laevis*	0.0E+00	121.3
HyS0002.129	CNOT10	Q5ZIW2.1	*G. gallus*	3.2E-146	50.5
HyS0002.409	CNOT11	Q9UKZ1.1	*H. sapiens*	4.7E-179	30.1
HyS0052.39	PARN	O95453.1	*H. sapiens*	1.0E-113	45.1

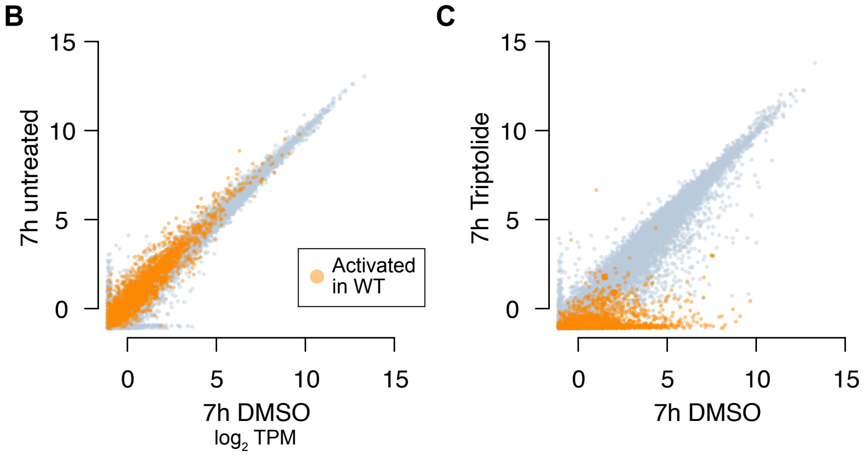

Supplementary Figure S5. Triptolide treatment inhibits genome activation. (A) Table of maternal expression levels of predicted deadenylase factors as identified by BLAST search. **(B)** Biplot showing expression levels in untreated versus DMSO vehicle embryos. Orange points are genes with significant activation over time in wild-type embryos. **(C)** Biplot showing failed activation of wild-type activated genes (orange) with Triptolide treatment. TPM = transcripts per million.

bioRxiv preprint doi: https://doi.org/10.1101/2023.05.09.540083; this version posted May 10, 2023. The copyright holder for this preprint (which was not certified by peer review) is the author/funder, who has granted bioRxiv a license to display the preprint in perpetuity. It is made available under aCC-BY 4.0 International license.

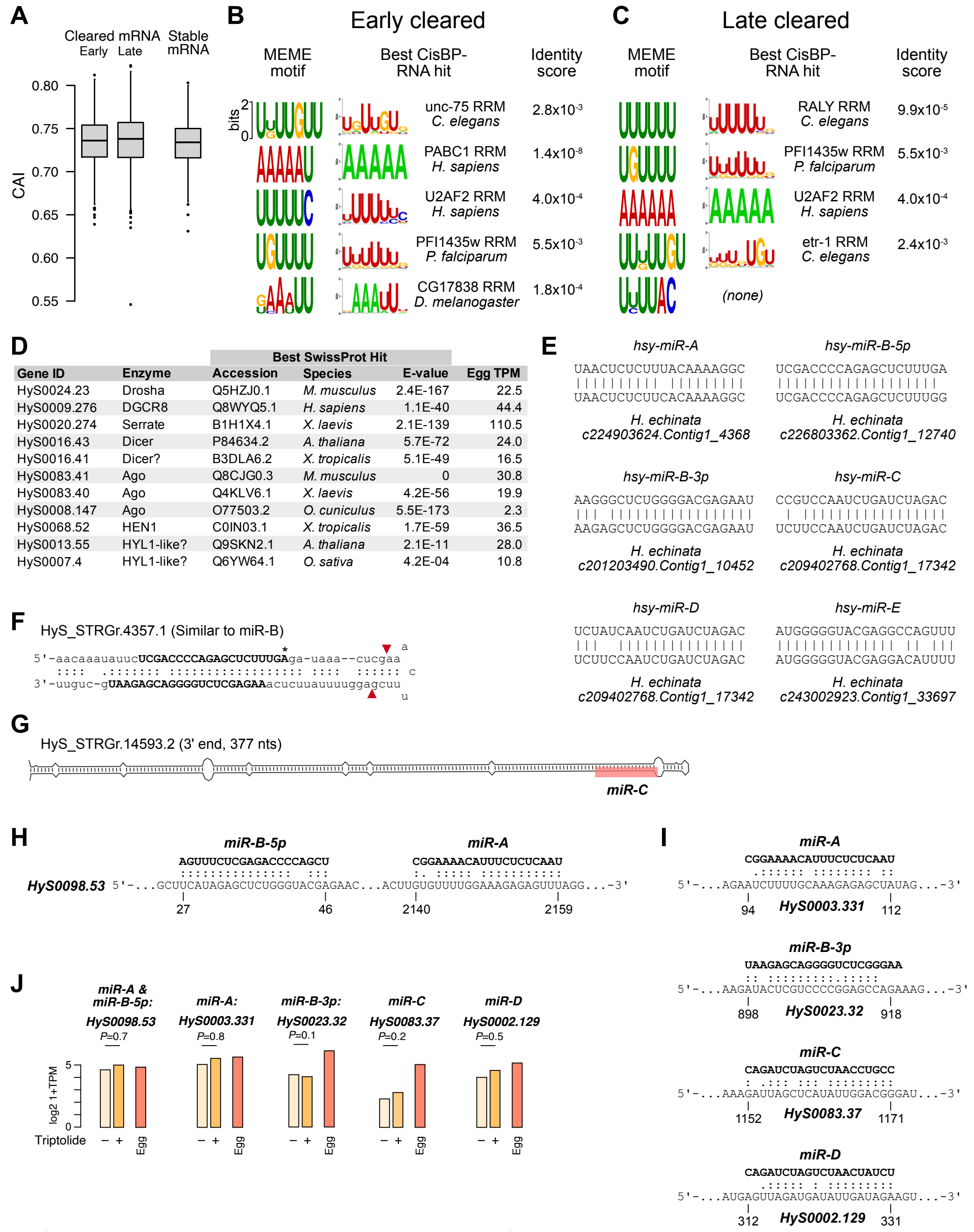

Gene ID	Enzyme	Accession	Species	E-value	Egg TPM
		Best SwissProt Hit			
HyS0024.23	Drosha	Q5HZJ0.1	*M. musculus*	2.4E-167	22.5
HyS0009.276	DGCR8	Q8WYQ5.1	*H. sapiens*	1.1E-40	44.4
HyS0020.274	Serrate	B1H1X4.1	*X. laevis*	2.1E-139	110.5
HyS0016.43	Dicer	P84634.2	*A. thaliana*	5.7E-72	24.0
HyS0016.41	Dicer?	B3DLA6.2	*X. tropicalis*	5.1E-49	16.5
HyS0083.41	Ago	Q8CJG0.3	*M. musculus*	0	30.8
HyS0083.40	Ago	Q4KLV6.1	*X. laevis*	4.2E-56	19.9
HyS0008.147	Ago	O77503.2	*O. cuniculus*	5.5E-173	2.3
HyS0068.52	HEN1	C0IN03.1	*X. tropicalis*	1.7E-59	36.5
HyS0013.55	HYL1-like?	Q9SKN2.1	*A. thaliana*	2.1E-11	28.0
HyS0007.4	HYL1-like?	Q6YW64.1	*O. sativa*	4.2E-04	10.8

Supplementary Figure S6. Evaluation of different potential maternal clearance mechanisms. (A) Comparison of codon adaptation index (CAI) between cleared mRNA and a set of stable mRNA, showing no significant decreased CAI associated with clearance. **(B, C)** Top hits from the CISBP-RNA database for each of the MEME motifs enriched in cleared genes. **(D)** Maternal expression levels for predicted components of the miRNA biogenesis pathway and the RNA-induced silencing complex. **(E)** Alignment of predicted *H. symbiolongicarpus* mature miRNAs with *H. echinata* miRNAs. **(F)** A second transcript that contains the predicted miR-B miRNA sequences, but in a structural context inconsistent with canonical Dicer processing (canonical Dicer cleavage sites marked by red arrows). Asterisk marks a base difference compared to *H. echinata*. **(G)** The full duplex structure in which predicted miRNA miR-C is found, suggesting it is not a Drosha substrate. **(H, I)** Predicted mRNA targets, as recovered by BLAST sequence similarity, showing potential base pairing configuration with the mature miRNAs. **(J)** RNA-seq expression levels of predicted mRNA targets of miRNAs in the presence or absence of Triptolide, as compared to maternal levels in the egg. None of the differences are significant, by DESeq2 with FDR adjustment.

bioRxiv preprint doi: https://doi.org/10.1101/2023.05.09.540083; this version posted May 10, 2023. The copyright holder for this preprint (which was not certified by peer review) is the author/funder, who has granted bioRxiv a license to display the preprint in perpetuity. It is made available under aCC-BY 4.0 International license.

Supplementary Table 1 - Oligos for rRNA-depletion
Supplementary Table 2 - RNA-seq expression levels
Supplementary Table 3 - Activated genes
Supplementary Table 4 - H4K20me transferase gene domains
Supplementary Table 5 - Stringtie-predicted genes
Supplementary Table 6 - Stage-specific genes
Supplementary Table 7 - Maternal clearance categories
